# Dieckol Attenuated Glucocorticoid-Induced Muscle Atrophy by Decreasing NLRP3 Inflammasome and Pyroptosis

**DOI:** 10.3390/ijms22158057

**Published:** 2021-07-28

**Authors:** Seyeon Oh, Jinyoung Yang, Chulhyun Park, Kukhui Son, Kyunghee Byun

**Affiliations:** 1 Functional Cellular Networks Laboratory, Department of Medicine, Graduate School, Lee Gil Ya Cancer and Diabetes Institute, Gachon University, Incheon 21999, Korea; seyeon8965@gmail.com (S.O.); roswellgirl111@gmail.com (J.Y.); 2 Department of Thoracic and Cardiovascular Surgery, Gachon University Gil Medical Center, Gachon University, Incheon 21565, Korea; cdgpch@gilhospital.com; 3 Department of Anatomy & Cell Biology, Gachon University College of Medicine, Incheon 21936, Korea

**Keywords:** RAGE, TLR 4, NLRP3 inflammasome, pyroptosis, *Ecklonia cava* extract, dieckol

## Abstract

Dexamethasone (Dexa), frequently used as an anti-inflammatory agent, paradoxically leads to muscle inflammation and muscle atrophy. Receptor for advanced glycation end products (RAGE) and Toll-like receptor 4 (TLR4) lead to nucleotide-binding oligomerization domain-like receptor pyrin domain containing 3 (NLRP3) inflammasome formation through nuclear factor-κB (NF-κB) upregulation. NLRP3 inflammasome results in pyroptosis and is associated with the Murf-1 and atrogin-1 upregulation involved in protein degradation and muscle atrophy. The effects of *Ecklonia cava* extract (ECE) and dieckol (DK) on attenuating Dexa-induced muscle atrophy were evaluated by decreasing NLRP3 inflammasome formation in the muscles of Dexa-treated animals. The binding of AGE or high mobility group protein 1 to RAGE or TLR4 was increased by Dexa but significantly decreased by ECE or DK. The downstream signaling pathways of RAGE (c-Jun N-terminal kinase or p38) were increased by Dexa but decreased by ECE or DK. NF-κB, downstream of RAGE or TLR4, was increased by Dexa but decreased by ECE or DK. The NLRP3 inflammasome component (NLRP3 and apoptosis-associated speck-like), cleaved caspase -1, and cleaved gasdermin D, markers of pyroptosis, were increased by Dexa but decreased by ECE and DK. Interleukin-1β/Murf-1/atrogin-1 expression was increased by Dexa but restored by ECE or DK. The mean muscle fiber cross-sectional area and grip strength were decreased by Dexa but restored by ECE or DK. In conclusion, ECE or DK attenuated Dexa-induced muscle atrophy by decreasing NLRP3 inflammasome formation and pyroptosis.

## 1. Introduction

Muscle atrophy is accompanied by several pathological conditions, such as cachexia, starvation, diabetes, metabolic acidosis, sepsis, and chronic kidney disease, while circulating glucocorticoid (GC) levels are elevated in these diseases [1]. High doses or long-term use of synthetic GCs, such as dexamethasone (Dexa), induce muscle atrophy [2]. Skeletal muscle plays various essential roles in energy metabolism, the release of various myokines, and human locomotion [3]. An extreme decrease of muscle mass leads to disability in physical function and increases the risk of morbidity and mortality [4]. Receptor for advanced glycation end products (RAGE) is a member of the immunoglobulin superfamily [5] and mediates several pathological conditions [6]. Advanced glycation end products (AGEs), the ligand of RAGE, is generated by the nonenzymatic glycation of proteins or lipids by exposure to reducing sugars [5]. AGEs formation is stimulated by enhanced oxidative stress conditions, such as hyperglycemia [7,8].

High mobility group protein 1 (HMGB1) and S100 calcium-binding protein are also ligands of RAGE [9,10].

Accumulation or increased expression of RAGE involves pathophysiological conditions of skeletal muscle or muscle wasting related to aging, inflammatory conditions, cancers, and metabolic disorders [6]. The AGE accumulation in the skeletal muscle of older people is increased compared to the young and relates to the deterioration of muscle function with aging [11,12].

By binding ligands, RAGE activates several downstream signaling pathways, including extracellular signal-regulated kinase 1/2, p38 mitogen-activated protein kinase, and c-Jun N-terminal kinase (JNK), and consequently leads to nuclear factor-κB (NF-κB) upregulation [13,14]. By NF-κB upregulation, the production of various proinflammatory cytokines, such as interleukin (IL)-6, IL-1β, and tumor necrosis factor-α, is increased [15]. 

Nucleotide-binding oligomerization domain-like receptor pyrin domain containing 3 (NLRP3), which produces inflammasome, is also activated by AGEs [16,17]. By binding AGEs to RAGE, JNK and p38 are activated, leading to NF-κB upregulation and NLRP3 inflammasome formation [18].

NLRP3 inflammasome is a complex of NLRP3, apoptosis-associated speck-like (ASC), and procaspase-1 [19]. NLRP3 inflammasome cleaves procaspase-1 as an activated form of caspase-1, leading to gasdermin D (GSDMD) cleavage [20]. The cleaved GSDMD forms a pore in the cellular membrane and induces pyroptosis, a form of cell death [20].

Pyroptosis, different from apoptosis and necrosis, is initiated by microbial infection or damage-associated molecular pattern (DAMP)-induced inflammation [21]. In addition, cleaved caspase-1, formed by NLRP3 inflammasome, enhances the maturation of proinflammatory cytokines, such as IL-1β [19].

Proinflammatory cytokines upregulated by NLRP3 inflammasome are related to muscular tissue wasting and atrophy [22]. HMGB1, one of the DAMPs, is also increased by NLRP3-mediated inflammasome formation through HMGB1-Toll-like receptor 4 (TLR4) signaling [23]. In addition, AGEs lead to TLR4 upregulation [24].

NF-κB expression is increased in human skeletal muscle in response to GC treatment [25]. Dexa increases the NF-κB expression in skeletal muscle [26]. NF-κB upregulation is accompanied by an increased expression of skeletal muscle atrogenes, such as atrogin-1 and muscle RING finger protein-1 (Murf-1), which induce skeletal muscle proteolysis [25].

NLRP3 inflammasome is involved in muscle atrophy, which is induced by systemic inflammation including sepsis [22]. In addition, GC treatment induced increasing NLRP3 inflammasome and thus led to decreased C2C12 myotube formation [27]. Dexa injection to C57BL/6J mice for 10 days also led to increasing pyroptosis and led to decreased myofiber cross sectional area of the gastrocnemius muscle [27].

Ecklonia is a genus of brown algae that belongs to the family Lessoniaceae and contains enriched eckol type phlorotannins [28]. Ecklonia has nine species and *Ecklonia cava*, an edible seaweed, has been used as food ingredient, animal feed, and fertilizer, as well as being used for production of fucoidan and phlorotannin [28].

Extracts of *Ecklonia cava* (ECE) have been reported to show antioxidant [29], anti-inflammatory [30], anti-diabetic [31], hepatoprotective [32], neuroprotective [33], antibacterial/antiviral [34], and anti-obesity effects [35].

Four compounds of phlorotannins, including dieckol (DK), phlorofucofuroeckol A, pyrogallol phloroglu-cinol-6,6-bieckol, and 2,7-phloroglucinol-6,6-bieckol have been isolated from ECE [36]. DK from ECE has been reported to show anti-diabetic [37], hepatoprotective [38], anti-obesity [39], anti-hypertensive [40], and anti-inflammatory effects [41].

In addition, it was shown that DK decreased TLR4 and RAGE expression in tubular epithelial cells exposed to hypoxic-reperfusion injury [42].

This study evaluated the effects of ECE and DK on attenuating Dexa-induced muscle atrophy by decreasing NLRP3 inflammasome formation. It was hypothesized that ECE or DK decreases RAGE and TLR4 expression, which consequently results in decreased NF-κB expression. Decreased NF-κB activity attenuates NLRP3 inflammasome formation, which consequently decreases the expression of muscle atrogenes.

## 2. Results

### 2.1. ECE and DK Decreased Bindings between RAGE/TLR4 and Their Ligands in Muscle of Dexa-Treated Animals

**Figure 1:**
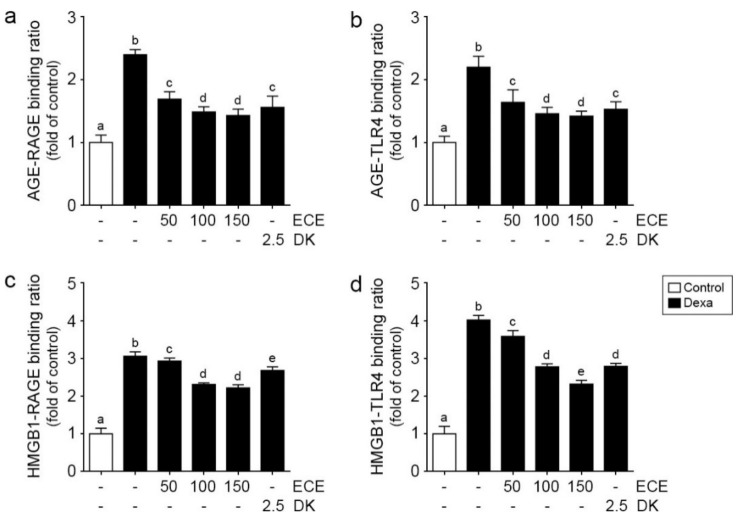
Inhibitory effects of ECE and DK on the relative binding levels of RAGE-AGE, RAGE-HMGB1, TLR4-AGE, and TLR4-HMGB1 in the gastrocnemius muscle of the Dexa-induced muscle atrophy model. The relative binding levels of RAGE-AGE (**a**), RAGE-HMGB1 (**b**), TLR4-AGE (**c**), and TLR4-HMGB1 (**d**) were increased by Dexa but decreased by ECE and DK treatment. The dose unit of ECE and DK is mg/kg/day. a–e: Different letters indicate significant differences among groups as determined by multiple comparison (Mann–Whitney U test); *p* < 0.05. AGE, advanced glycation end products; Dexa, dexamethasone; DK, dieckol; ECE, *Ecklonia cava* extract; HMGB1, high mobility group box 1; RAGE, receptor of AGE; TLR4, toll like receptor 4.

The binding ratio between AGE and RAGE in the muscle in Dexa-treated animals was significantly higher than age-matched control animals and significantly decreased by ECE or DK administration. The decreasing effect was most prominent at 100 and 150 mg/kg ECE (Figure 1a).

The binding ratio between AGE and TLR4 was significantly increased by Dexa treatment and significantly decreased by ECE or DK administration. The decreasing effect was most prominent at 100 and 150 mg/kg ECE (Figure 1b).

The binding ratio between HMGB1 and RAGE was significantly increased by Dexa treatment and significantly decreased by ECE and DK administration. The decreasing effect was most prominent at 100 and 150 mg/kg ECE (Figure 1c).

The binding ratio between HMGB1 and TLR4 was significantly increased by Dexa treatment and significantly decreased by ECE or DK administration. The decreasing effect was most prominent at 150 mg/kg ECE (Figure 1d).

### 2.2. ECE and DK Decreased JNK/p38 Activity and NF-κB Expression

**Figure 2:**
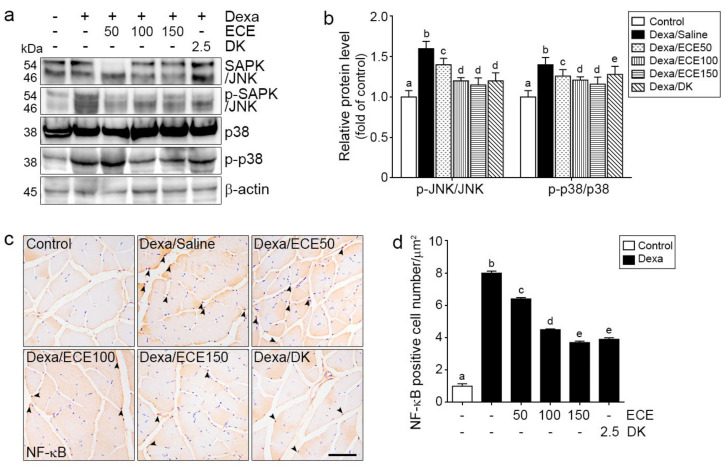
Inhibitory effects of ECE and DK on NF-κB activation in the gastrocnemius muscle of the Dexa-induced muscle atrophy model. (**a**,**b**) Protein levels of NF-κB activation-related molecules (SAPK/JNK and p38) were determined by Western blotting. As a result, the ratios of p-SAPK/JNK to JNK and p-p38 to p38 were increased by Dexa but decreased by ECE and DK treatment. (**c**,**d**) The amount of NF-κB translocated into the nucleus was increased by Dexa but decreased by ECE and DK treatment. The dose unit of ECE and DK is mg/kg/day. a–e: Different letters indicate significant differences among groups, as determined by multiple comparison (Mann–Whitney U test); *p* < 0.05. Dexa, dexamethasone; DK, dieckol; ECE, *Ecklonia cava* extract; NF-κB, nuclear factor kappa-light-chain-enhancer of activated B cells; SAPK/JNK, stress-activated protein kinase/Jun-amino-terminal kinase; p-SAPK/JNK, phospho-SAPK/JNK; p-p38, phopho-p38.

The ratio of pSAPK/JNK and SAPK/JNK in the muscle was significantly increased by Dexa treatment and significantly decreased by ECE or DK administration. The decreasing effect was most prominent at 100 and 150 mg/kg ECE and DK (Figure 2a,b).

The ratio of p-p38 and p38 in the muscle was significantly increased by Dexa treatment and significantly decreased by ECE or DK administration. The decreasing effect was most prominent at 100 and 150 mg/kg ECE (Figure 2a,b).

NF-κB expression in the nuclei was significantly increased by Dexa treatment and significantly decreased by ECE or DK administration. The decreasing effect was most prominent at 150 mg/kg ECE and DK (Figure 2c,d). 

### 2.3. ECE and DK Attenuated NLRP3 Inflammasome Formation and Pyroptosis in the Muscle of Dexa-Treated Animals

**Figure 3:**
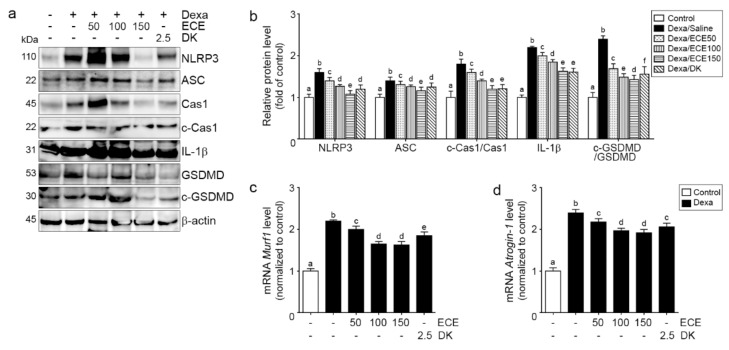
Effects of ECE and DK on pyroptosis by reducing NLRP3 inflammasome formation in the gastrocnemius muscle of the Dexa-induced muscle atrophy model. (**a**,**b**) Protein levels of NLRP3 inflammasome-pyroptosis-related molecules (NLRP3, ASC, Cas1, c-Cas1, IL-1β, GSDMD, and cleaved GSDMD) were determined by Western blotting. As a result, NLRP3 inflammasome-pyroptosis-related molecules were increased by Dexa but decreased by ECE and DK. (**c**,**d**) mRNA levels of *Murf1* and *Atrogin-1*, related to muscle protein degradation, were increased by Dexa but decreased by ECE and DK. The dose unit of ECE and DK is mg/kg/day. a–f: Different letters indicate significant differences among groups, as determined by multiple comparison (Mann–Whitney U test); *p* < 0.05. ASC, adaptor molecule apoptosis-associated speck-like protein containing CARD; Cas1, caspase 1; c-Cas1, cleaved-caspase 1; c-GSDMD, cleaved-gasdermin D; Dexa, dexamethasone; DK, dieckol; ECE, *Ecklonia cava* extract; GSDMD, gasdermin D; IL-1β, interleukin-1beta; NLRP3, NOD-like receptor pyrin domain-containning protein 3.

NLRP3 and ASC expression was significantly increased by Dexa and significantly decreased by ECE or DK administration. The decreasing effect was most prominent at 150 mg/kg ECE (Figure 3a,b).

The ratio of cleaved caspase-1 and caspase-1 was significantly increased by Dexa and significantly decreased by ECE or DK administration. The decreasing effect was most prominent at 150 mg/kg ECE and DK (Figure 3a,b).

IL-1β expression was significantly increased by Dexa and significantly decreased by ECE or DK administration. The decreasing effect was most prominent at 150 mg/kg ECE and DK (Figure 3a,b).

The ratio of cleaved GSDMD and GSDMD was significantly increased by Dexa and significantly decreased by ECE or DK administration. The decreasing effect was most prominent at 150 mg/kg ECE (Figure 3a,b).

Murf-1 and atrogin-1 expression was significantly increased by Dexa and significantly decreased by ECE or DK administration. The decreasing effect was most prominent at 100 and 150 mg/kg ECE (Figure 3c,d).

### 2.4. ECE and DK Attenuated Muscle Atrophy and Improved Grip Strength

**Figure 4:**
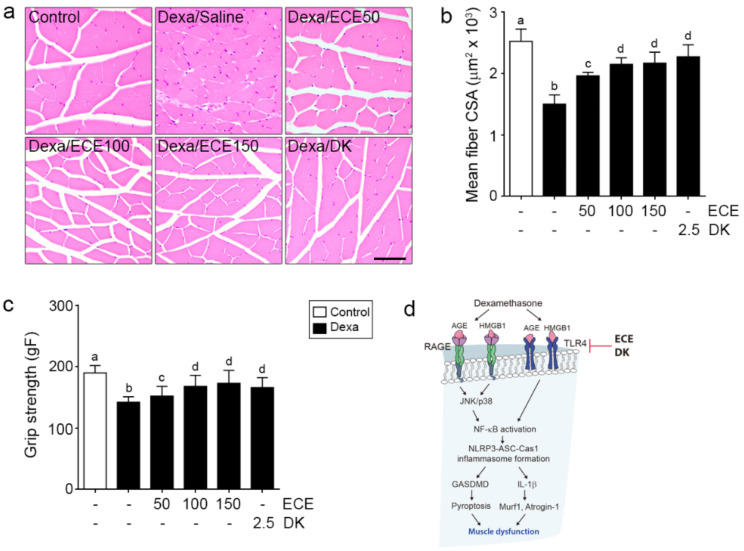
Effects of ECE or DK on recovering muscle strength by reducing pyroptosis in the gastrocnemius muscle of the Dexa-induced muscle atrophy model. (**a**) Representative image of H&E stained cross-sections of the gastrocnemius muscle. (**b**) The mean muscle fiber CSA was decreased by Dexa but increased by ECE and DK. (**c**) Grip strength in the study groups before sacrifice. Grip strength was decreased by Dexa but increased by ECE and DK. (**d**) Summary. The dose unit of ECE and DK is mg/kg/day. a–d: Different letters indicate significant differences among groups as determined by multiple comparison (Mann–Whitney U test); *p* < 0.05. CSA, cross-sectional area; Dexa, dexamethasone; DK, dieckol; ECE, *Ecklonia cava* extract.

The mean cross-sectional area (CSA) of muscle fibers was significantly decreased by Dexa and significantly increased by ECE and DK administration. The increasing effect was most prominent at 100 mg/kg and 150 mg/kg ECE and DK (Figure 4a,b).

Grip strength was significantly decreased by Dexa and significantly increased by ECE and DK administration. The increasing effect was most prominent at 100 mg/kg and 150 mg/kg ECE and DK (Figure 4c). 

## 3. Discussion

Although GCs are representative anti-inflammatory and immunosuppressive agents [43], they also stimulate inflammatory signaling pathways that lead to muscle atrophy [44].

Pyroptosis is a type of proinflammatory programmed cell death and has specific characteristics, in which GSDMD cleavage forms cytotoxic pores in the cell membrane [45]. The most well-known upstream activator of pyroptosis is NLRP3, and NLRP3 inflammasome enhances caspase-1-dependent pyroptosis, leading to the release of the mature form of inflammatory factors, such as IL-1β and IL-18 [46]. Pyroptosis is also known to be involved in the pathophysiology of muscle atrophy [47].

Dexa enhanced NLRP3, caspase-1, and GSDMD expression in C2C12 myotubes, and NLRP3 or GSDMD knockdown decreased Dexa-induced myotube pyroptosis [27].

The signaling pathway of RAGE/JNK or p38, which stimulates the downstream signaling pathway of NF-κB, is known to increase NLRP3 inflammasome formation [18].

HMGB1/TLR4/NF-κB signaling is also related to the upregulation of NLRP3 inflammasome formation [23].

This study showed that the binding of AGEs or HMGB1 to RAGE or TLR4 in skeletal muscle was increased by Dexa, as well as the binding of HMGB1 to RAGE, binding of AGE to TLR4, binding of AGE to RAGE, and binding of HMGB1 to TLR4 (Figure 1). The downstream signaling pathway of RAGE, activated JNK, and p38 was significantly increased by Dexa. NF-κB expression, upregulated by RAGE/JNK or p38 and TLR4, was significantly increased by Dexa. ECE and DK decreased binding of AGE or HMGB1 to RAGE or TLR4, and this decreased binding contributed to decreasing the RAGE/JNK, p38/NF-κB, or TLR4/NF-κB pathways (Figure 2).

Increased expression of caspase-1, secretion of IL-1β (facilitated by caspase-1), and increased substrate of GSDMD, are frequently reported in bacterial infection-related pyroptosis [48] and sterile inflammation [49]. Thus, these factors were used as markers of pyroptosis [23]. Diabetes, a sterile inflammatory condition, is related to increased pyroptosis, characterized by increased caspase-1, IL-1β, and GSDMD in skeletal muscle [23].

This study showed that the expression of NLRP3 and ASC, the components of NLRP3 inflammasome, was increased by Dexa. Furthermore, cleaved caspase-1, IL-1β, and cleaved GSDMD were increased by Dexa (Figure 3a,b). Increased IL-1β by NLRP3 inflammasome leads to the upregulation of atrogenes [22]. This study showed that increased IL-1β was related to increased Murf1 and Atrogin-1 expression. Murf1 and Atrogin-1 expression was increased by Dexa (Figure 3c,d). ECE and DK attenuated the expression of NLRP3/ASC, cleaved caspase-1, and cleaved GSDMD, which were increased by Dexa (Figure 3a,b). In addition, ECE and DK attenuated IL-1β and Murf-1 and Atrogin-1 expression (Figure 3c,d). Murf1 and atrogin-1 are part of the ubiquitin-proteasome pathway, the principal pathway of intracellular protein degradation in skeletal muscle [50].

This study showed that the mean muscle fiber CSA and grip strength were decreased by Dexa but restored by ECE and DK administration (Figure 4). Dexa is a widely used agent for various clinical conditions and paradoxically increases mortality during treatment by increasing muscle atrophy. Thus, it is essential to find ways to minimize muscle atrophy during Dexa use.

These results suggested that ECE or DK could attenuate Dexa-induced muscle atrophy by modulating NRLP3 inflammasome and pyroptosis (Table S1).

## 4. Materials and Methods

### 4.1. Preparation of ECE and Isolation of DK

*Ecklonia cava* was obtained from Aqua Green Technology Co., Ltd. (Jeju, Republic of Korea). For extraction, *Ecklonia cava* was washed and air-dried at room temperature for 48 h, after which the blades were ground and 50% ethanol was added, followed by incubation at 85 *°*C for 12 h. ECE was filtered, concentrated, sterilized by heating to ≥85 °C for 40–60 min, and then spray-dried. DK, one of the representative phlorotannins in ECE, was isolated using centrifugal partition chromatography (CPC). CPC was performed using a two-phase solvent system comprising water/ethyl acetate/methyl alcohol/n-hexane (7:7:3:2, *v*/*v*/*v*/*v*). The organic stationary phase was filled in the CPC column, followed by pumping of the mobile phase into the column in descending mode at the same flow rate used for separation (2 mL/min). The purity of DK used in the study was confirmed to be 93.58% [36].

### 4.2. Dexa-Induced Muscle Atrophy Model

Adult CrljOri:CD1 (ICR) mice (male, aged 9 weeks, 33~36 g) were obtained from OrientBio Inc. (Seungnam, Repuvlic of Korea). The animals were routinely cared for under controlled environmental conditions (temperature of ~23 °C, relative humidity of 50%, and a light/dark cycle 12 h/12 h) with ad libitum access to water and a standard laboratory rodent chow diet. After 1 week of adaption period, mice were randomly categorized into six groups of 3 mice in each group: (i) the saline treated group (Control); (ii) the saline treated Dexa induced muscle atrophy group (Dexa/Saline); (iii) the 50 mg/kg of ECE treated Dexa induced muscle atrophy group (Dexa/ECE50); (iv) the 100 mg/kg of ECE treated Dexa induced muscle atrophy group (Dexa/ECE100), (v) the 150 mg/kg of ECE treated Dexa induced muscle atrophy group (Dexa/ECE150), and (vi) the 2.5 mg/kg of DK treated Dexa induced muscle atrophy group (Dexa/DK). ECE or DK was dissolved in saline and the control group was given the same amount of saline water. ECE or DK were administered by gavage once a day for 28 days prior to Dexa injection and maintained throughout the experimental period of 38 days. The muscle atrophy model applied in this study was to induce muscle atrophy by subcutaneous injection of Dexa (1 mg/kg) once a day for 10 days [51,52] (Figure S1). All animal experiments were conducted with approval and according to the ethical principles of the Institutional Animal Care and Use Committee of Gachon University (approval no. LCDI-2020-0030).

### 4.3. Sample Preparation

#### 4.3.1. Protein Isolation

Gastrocnemius muscle proteins were extracted using a EzRIPA lysis kit (ATTO, Tokyo, Japan). Initially, tissue was homogenized with lysis buffer containing proteinase and phosphatase inhibitors and briefly sonicated for 10 s in a cold bath sonicator. After centrifugation at 14,000*× g* for 20 min at 4 °C, the supernatants were collected, and protein concentrations were determined using a bicinchoninic acid assay kit (BCA kit; Thermo Fisher Scientific, Waltham, MA, USA).

#### 4.3.2. RNA Extraction and cDNA Synthesis

Gastrocnemius muscle was homogenized in ice using a disposable pestle in 1 mL RNiso (Takara, Tokyo, Japan). The homogenates were added to 0.2 mL chloroform, mixed, and centrifuged at 12,000*× g* for 15 min at 4 °C. The aqueous phase was collected, placed in cleaned tubes, mixed with 0.5 mL isopropanol, and centrifuged at the same conditions. The supernatant was discarded, leaving only the RNA pellet, which was then washed with 70% ethanol and dissolved in 50 µL diethyl pyrocarbonate-treated water. The isolated RNA was synthesized with cDNA using a Prime Script First-Strand cDNA Synthesis Kit, according to the manufacturer’s instructions (Takara).

### 4.4. Sandwich Enzyme-Linked Immunosorbent Assay (Sandwich ELISA)

The 96-well microplates were coated with anti-RAGE and anti-TLR4 antibodies (listed in Table S1) diluted in 100 nM carbonate and bicarbonate mixed buffer, adjusted to pH 9.6, and incubated overnight at 4 °C. The microplates were then washed with phosphate-buffered saline (PBS) containing 0.1% Triton X-100 (TPBS). The remaining protein-binding sites were then blocked using 5% skim milk at 4 °C overnight. After washing with TPBS, the gastrocnemius protein samples were distributed to each well and incubated for 90 min at 37 °C. After washing the plate with TPBS, 1 μg/mL of anti-AGE or anti-HMGB1 antibodies (listed in Table S1) was added for 4 h at room temperature. Each well was rinsed with TPBS and then incubated for 2 h at room temperature with a peroxidase-conjugated secondary antibody. Tetramethylbenzidine solution was added, followed by incubation for 15 to 20 min at room temperature. Sulfuric acid (2 N) was used as a stop solution. Optical density was measured at a wavelength of 450 nm using a microplate reader (Spectra Max Plus; Molecular Devices, San Jose, CA, USA). 

### 4.5. Westerm Blotting

Protein lysates were prepared as described above. Equal amounts of proteins were separated by 10% sodium dodecyl sulfate-polyacrylamide gel electrophoresis and then transferred to polyvinylidene fluoride membranes, which were incubated with the appropriate diluted primary antibodies at 4 °C overnight (listed in Table S1). Membranes were then washed with Tris-buffered saline containing 1% Tween 20 thrice and incubated with secondary antibodies for 1 h at room temperature. Membranes were developed by enhanced chemiluminescence on LAS-4000s (GE Healthcare, Chicago, IL, USA).

### 4.6. Immunohistochemistry

Tissue blocks of paraffin-embedded gastrocnemius muscle were cut into 7-µm-thick sections, placed on a coated slide, and dried at 45 °C for 24 h. Slides were deparaffinized and incubated in normal animal serum to block nonspecific antibody binding and then incubated with primary antibodies (listed in Table S1) at 4 °C, followed by three additional rinses with PBS. Slides were treated with biotinylated secondary antibodies using an ABC kit (Vector Laboratories, Burlingame, CA, USA), incubated for 1 h with secondary antibody solution, and rinsed thrice with PBS. Slides were left to react with 3,3′-diaminobenzidine substrate for up to 15 min, followed by mounting with a coverslip and DPX mounting solution (Sigma-Aldrich, St. Louis, MO, USA). Images were obtained using a light microscope (Olympus, Tokyo, Japan), and the intensity of the brown color was quantified using ImageJ software (National Institutes of Health, Bethesda, MD, USA).

### 4.7. Quantitative Real-Time Polymerase Chain Reaction (qRT-PCR)

qRT-PCR was performed using cDNA synthesis with a CFX384 Touch^TM^ Real-time PCR detection system. Then, 300 ng cDNA, 5 μL SYBR premix (Takara), and 0.4 μM forward and reverse primers (listed in Table S2) were mixed and the threshold cycle numbers were determined using CFX Manager^TM^ software.

### 4.8. Hematoxylin and Eosin (H&E) Staining

H&E staining was used to determine the muscle fiber cross-sectional area (CSA). Gastrocnemius muscle tissue slides were incubated with hematoxylin (DAKO, Glostrup, Denmark) for 1 min, rinsed in distilled water for 10 min, and placed in eosin Y solution (Sigma-Aldrich) for 1 min at room temperature. Nuclei were detected with blue color, and cytoplasm was detected with light pink. The completed slides were observed under an optical microscope (Olympus Optical Co., Nagano, Japan). The mean muscle fiber CSA was measured by ImageJ software. Histological analyses were conducted in a blinded manner, and three operators conducted each analysis at least in triplicate.

### 4.9. Statistical Analysis

Nonparametric tests were performed in this study. A Kruskal–Wallis test was used to determine the significance of differences among the 6 groups. If a significant difference was confirmed by the Kruskal–Wallis test, multiple comparisons for 2 groups were performed with a Mann–Whitney U-test. The results were presented as mean ± standard deviation and statistical significance was accepted for *p* < 0.05. Statistical significance was accepted as follows. Statistical analysis was performed using SPSS version 22 (IBM Corporation, Armonk, NY, USA).

## 5. Conclusions

In conclusion, ECE or DK decreases the signaling pathways of RAGE/JNK or p38 and HMGB1/TLR4, eventually leading to decreased NF-κB expression in Dexa-induced muscle atrophy. ECE or DK also decreases NLRP3 inflammasome formation and pyroptosis, leading to decreased IL-1β/Murf-1/atrogin-1 expression in Dexa-induced atrophy (Figure 4d).

## Data Availability

All data is contained within the article.

## Supplementary Materials

The following are available online at https://www.mdpi.com/article/10.3390/ijms22158057/s1.
