# Supplementary material for: Dieckol Attenuated Glucocorticoid-Induced Muscle Atrophy by Decreasing NLRP3 Inflammasome and Pyroptosis
Source: Int J Mol Sci. 2021 Jul 28;22(15):8057. doi: 10.3390/ijms22158057 (PMC8348567; doi:10.3390/ijms22158057)
Supplement: Supplementary file 1 [file ijms-22-08057-s001.zip › ijms-1313292-supplementary.pdf]

Dieckol Attenuated Glucocorticoid-Induced Muscle Atrophy by Decreasing NLRP3 Inflammasome and Pyroptosis

Seyeon Oh [1], Jin Young Yang [1], Kuk Hui Son [2,*] and Kyunghee Byun [2,*]

[1] Functional Cellular Networks Laboratory, College of Medicine, Department of Medicine, Graduate School and Lee Gil Ya Cancer and Diabetes Institute, Gachon University, Incheon 21999, Republic of Korea; seyeon8965@gmail.com (S.O.); roswellgirl111@gmail.com (J.Y.Y.)

[2] Department of Thoracic and Cardiovascular Surgery, Gachon University Gil Medical Center, Gachon University, Incheon 21565, Republic of Korea

* Correspondence: dr632@gilhospital.com; Tel.: +82-32-460-3666, khbyun1@gachon.ac.kr; Tel.: +82-32-899-6511

Supplementary Tables

Table S1. Results on the effects of ECE and DK in Dexa-induced muscle atrophy.

Results		Control	Dexa/ Saline	Dexa/ ECE50	Dexa/ ECE100	Dexa/ ECE150	Dexa/ DK
Binding ratio	AGE-RAGE	+	++++	+++	++	++	+++
	AGE-TLR4	+	++++	+++	++	++	+++
	HMGB1-RAGE	+	+++++	++++	++	++	+++
	HMGB1-TLR4	+	+++++	++++	+++	++	+++
NF-κB activation related molecules	pJNK	+	++++	+++	++	++	++
	pP38	+	+++++	+++	++	++	++++
	NF-κB	+	+++++	++++	+++	++	++
Pyroptosis related molecules	NLRP3	+	+++++	++++	+++	++	+++
	ASC	+	+++++	+++++	+++	++	+++
	c-Cas1	+	+++++	++++	+++	+	+
	IL-1β	+	+++++	++++	+++	++	++
	c-GSDMD	+	++++++	+++++	+++	++	++++

	**Murf1**	+	+++++	++++	++	++	+++
	**Atrogin-1**	+	++++	+++	++	++	+++
Muscle fiber size and function	**CSA**	++++	+	++	+++	+++	+++
	**Grip strength**	++++	+	++	+++	+++	+++

AGE, advanced glycation end products; ASC, adaptor molecule apoptosis-associated speck-like protein containing a CARD; Cas1, caspase 1; c-Cas1, cleaved-caspase 1; CSA, cross-sectional area; c-GSDMD, cleaved-gasdermin D; Dexa, dexamethasone; DK, dieckol; ECE, ecklonia cava extract; GSDMD, gasdermin D; HMGB1, high mobility group box 1; IL-1β, interleukin-1beta; NF-κB, nuclear factor kappa-light-chain-enhancer of activated B cells;NLRP3, NOD-like receptor pyrin domain-containning protein 3; RAGE, receptor of AGE; pP38, phopho-P38; p-SAPK/JNK, phospho-SAPK/JNK; SAPK/JNK, stress-activated protein kinase/Jun-amino-terminal kinase; TLR4, toll like receptor 4.

Table S2. List of antibodies for ELISA, Western blotting and immunohistochemistry used in this study.

Antibody name	Company	Dilution rate		
		ELISA	Western blotting	Immunohistochemistry
AGE	abcam	1:1,000		
HMGB1	abcam	1:1,000		
RAGE	Santa cruz Biotechnology	1:200		
TLR4	Novos Biological	1:500		
SAPK/JNK	Cell signaling		1:1,000	
p-SAPK/JNK	Cell signaling		1:1,000	
P38	Cell signaling		1:1,000	
pP38	Cell signaling		1:1,000	
NF-κB	Cell signaling			1:400
NLRP3	Abca,		1:500	
ASC	Santa cruz Biotechnology		1:1,000	
Cas1	Santa cruz Biotechnology		1:1,000	
c-Cas1	Cell signaling		1:1,000	
IL-1β	Santa cruz Biotechnology		1:500	
GSDMD	Cell signaling		1:1,000	
c-GSDMD	Cell signaling		1:1,000	
β-actin	Cell signaling		1:1,000	1:200

Table S3. List of primer for qRT-PCR used in this study.

Gene		Primers
actb	Forward	5'-ACA AAG CTG TTC AGT GTC TCC A-3′
	Reverse	5'-CTC CGT TTC CAG AAT ACA CAC A-3′
MuRF1	Forward	5'-ATC TAG CCT GAT TCC TGA TGG A-3′
	Reverse	5'-ACC ACA GGC TTG GTA AAC ATC T-3′
Atrogin-1	Forward	5'-GAC ATT CAG AAC AGC AAA ACC A-3′
	Reverse	5'-GCT CCT TCG TAC TTC CTT TGT G-3′

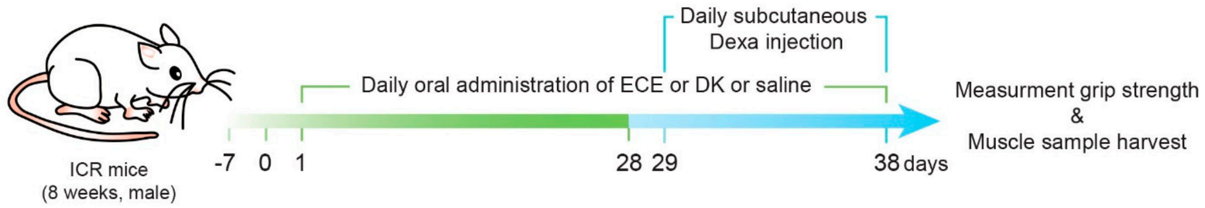

Figure S1. A schematic diagram of the animal model to confirm the effect of ECE or DK in Dexa-induced muscle atrophy. After 1 week of adaption period, ECE (50, 100 or 150 mg/kg) or DK (2.5 mg/kg) or saline were administrated by gavage once a day for 28 days prior to Dexa injection and maintained throughout the experimental period of 38 days. The muscle atrophy was induced by subcutaneous injection of Dexa (1mg/kg) once a day for 10 days. ECE, *Ecklonia cava* extract; DK, dieckol; Dexa; dexamethasone.
